# Efficacy of Homologous, Platelet-rich Plasma Dressing in Chronic Non-healing Ulcers: An Observational Study

**DOI:** 10.7759/cureus.2145

**Published:** 2018-02-02

**Authors:** Ravi Prabhu, Chellappa Vijayakumar, Asayas A Bosco Chandra, K Balagurunathan, Raja Kalaiarasi, K Venkatesan, Erabati Santosh Raja, Swetha T

**Affiliations:** 1 General Surgery, Sri Lakshmi Narayana Institute of Medical Science, Puducherry, India; 2 Surgery, Jawaharlal Institute of Postgraduate Medical Education and Research (JIPMER), Puducherry, India.; 3 Surgery, Sri Lakshmi Narayana Institute of Medical Science, Puducherry, India; 4 Otorhinolaryngology, Sri Lakshmi Narayana Institute of Medical Science, Puducherry, India; 5 Obstetric and Gynaecology, Mahatma Gandhi Medical College and Research Institute, Pondicherry, India

**Keywords:** platelets rich plasma, homologous, chronic ulcers, non healing ulcers

## Abstract

Background

Chronic non-healing ulcers are a frequent problem in developing countries and represent a heavy burden to the patients because they lack the necessary growth factors (GFs) to maintain the healing process and are frequently complicated by super, added infections. Traditional therapies, such as regular dressings and wound debridement, cannot provide satisfactory results since these treatments are not able to provide the necessary GFs. Platelet-rich plasma (PRP) helps in enhancing the wound healing by releasing various GFs. The aim was to evaluate the efficacy of PRP dressing in the treatment of chronic non-healing ulcers.

Methods

Patients attending the outpatient department on a regular basis and those admitted as inpatients for chronic wound management were included in the study. It was an observational study done in a tertiary health center for a period of one year. All patients with eligible criteria were treated with PRP at twice-weekly intervals for a maximum of 10 dressings. At the end of the five weeks period, the reduction in the size of the ulcers (area and volume) was assessed.

Results

A total of 104 cases with chronic non-healing ulcers of various causes were treated with homologous PRP twice weekly for a maximum of 10 dressings. In those 104 patients, non-healing ulcers in 85 patients (81.73%) were healed at the end of the last dressing. Non-healing ulcers in 13 patients (12.5%) were healed with skin grafting. Among those patients, the baseline mean ulcer area was 5.03 cm^2^. For each visit, there was a reduction in the ulcer area. At the end of the last visit, the mean ulcer area was 1.69 cm^2^, which was significant in this study.

Conclusion

Due to the lack of necessary GFs in chronic non-healing ulcers, PRP is safe and enhances the healing rates of chronic wounds, thereby reducing overall hospital stay and morbidity.

## Introduction

The loss of skin and subcutaneous tissue on the leg or foot that takes more than six weeks to heal is considered a chronic non-healing leg ulcer. This causes both physical and financial pain and discomfort [1]. Chronic non-healing ulcers lack the necessary growth factors (GFs) and the levels of proteases are high; hence, they do not heal well [2]. Wound healing is a complex and dynamic process of cellular, immunological, and hormonal components interacting to result in wound healing [3]. Most of the ulcers will eventually heal completely with appropriate wound care. But, this is affected by some of the risk factors associated with patient comorbidities or infections.

Platelet-rich plasma (PRP) is one of the new treatments for wound therapy for acute and chronic wounds. It is a platelet concentrate that has been widely used in a variety of clinical applications. A number of studies show that PRP enhances and accelerates both soft tissue and hard tissue healing. Autologous PRP gel consists of cytokines, GFs, chemokines, and fibrin derived from the patient’s blood [4].The mechanism of action for the PRP gel is thought to be the molecular and cellular induction of normal wound healing responses [5]. Autologous PRP is a safe, easy, and cost-effective method with good results in the management of chronic non-healing ulcers.

## Materials and methods

Study design

This observational study was conducted on 104 patients, either attending the outpatient department on a regular basis or admitted as inpatients for the management of chronic wounds in a tertiary care center for the period of one year.

Preparation of PRP

For the preparation of homologous PRP, 30 ml of donor blood was collected from the blood bank. The patient’s blood was cross-matched with donor blood to prevent local reactions. The blood was not chilled at any time before or during platelet separation. The blood was centrifuged at 2000 rpm for five minutes to obtain plasma. Then, this plasma was centrifuged at 3000 rpm for another five minutes to obtain a platelet concentrate. At the bottom of the tube, platelet pellets were formed. The lower 1/3rd is PRP and the rest of the plasma was considered as platelet poor plasma (PPP). After removing the PPP, the final product obtained was PRP.

Patient selection

This study included all patients in the 18-80-year age group, chronic non-healing ulcers ( ≥ 8 weeks), hemoglobin ( > 10 gm/dL), fasting blood sugar ≤ 110 mg% if non-diabetic, post-prandial blood sugar ≤ 140 mg% if diabetic, and sterile culture on wound swab. This study excluded patients with evidence of malignancy, active infection with pus discharge/slough, ulcer with exposed tendons/ligaments/bone, evidence of gangrene, and limb with selective Ankle Brachial Index (ABI ≥ 0.8 and ≤ 1.5). Patients with ulcers of Wagner’s grades III, IV, & V or Charcot’s foot and those who could not come for regular follow-up were excluded. The study also excluded patients who had received radiation or chemotherapy within the last three months. If the patient had a known or suspected case of osteomyelitis or was receiving steroids/antibiotics for another illness, they were also excluded from the study.

Written informed consent was obtained from all patients undergoing PRP application. Full-thickness skin or soft tissue defects were present in these patients. There was no necrotic tissue in the wound, for which the risk of infection was assumed to be low or removed by wound debridement. Ulcer types were divided into diabetic and non-diabetic. The hemoglobin of the patient should not have been below 11 gm/dL, with hematocrit above 35%, which was checked before the autologous transfusion. The patient should not have consumed acetaminophen, aspirin, or alcohol for 48 hours before transfusion.

PRP dressing application

**Figure 1:**
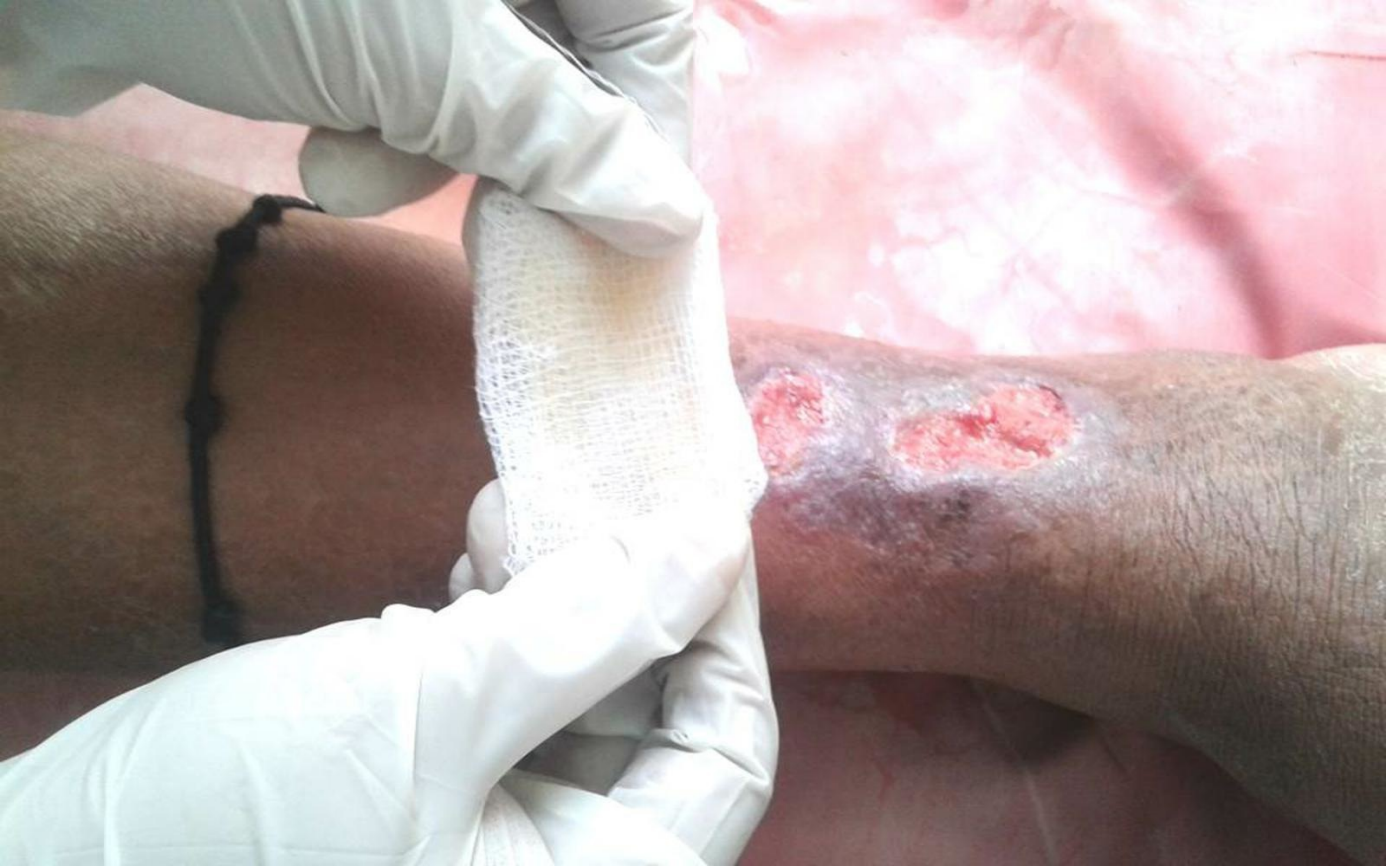
Platelet-rich plasma (PRP) dressing application

After debridement, the wound was irrigated with normal saline and dressed with sterile gauze impregnated with PRP and supported by two pieces of dry sterile gauze. The dressing was fixed using cotton bands. In an interval of five days, this method was used three times and regular saline dressing was applied as the shelf life of PRP is five to seven days. PRP dressing was repeated at a five-day interval for a maximum of 10 sittings of dressing (Figure 1).

Parameters

Along with baseline demographic parameters, primary outcome parameters, such as the size of the ulcer, grade of the ulcer, and wound surface area (in cm^2^), were assessed at the end of every week up to the fifth week. The percentage of the wound covered with granulation tissue, the percentage of wound covered with slough or nonviable tissue, and the time taken for the healing of the ulcer were measured using a visual score. The dressing is discontinued before five weeks if the ulcer is considered healed (complete re-epithelization of the ulcerated area) to the satisfaction of the treating surgeon. Secondary outcome parameters, such as patient acceptability of the PRP dressing, patient satisfaction with the treatment, and the healing of the ulcer, were also studied. 

## Results

A total of 104 cases with chronic non-healing ulcers of various causes were treated with homologous PRP twice weekly for a maximum of 10 dressings. The mean age of the patients was 52.34 with a body mass index (BMI) of 25.25 kg/m^2^. The mean hemoglobin (g/dL) and HbA1c were 11.78 and 6.2, respectively. According to the etiology, ulcers were classified as diabetic, venous, traumatic, decubitus ulcers, and others (postoperative wounds, Hansen’s disease), which constitutes 38.4%, 15.3%, 23.07, 19.2%, and 3.8%, respectively. Among the study, 84 (80.7%) were diabetic ulcers in which 70 patients (66.6%) were on oral hypoglycemic drugs and 34 patients (33.3%) were on insulin and eight (7.6%) were on antihypertensive drugs (Table 1).

**Table 1:** Baseline demographic parameter distribution in ulcer patients

DEMOGRAPHIC PARAMETER	NUMBER OF PATIENTS
Age (years)	52.34
Body Mass Index (kg/m^2^)	25.25
Hemoglobin (g/dL)	11.78
HbA1c	6.2
Comorbidities	
Diabetes Mellitus	84 (80.7%)
Hypertension	8 (7.6%)
TYPE OF ULCER	
Diabetic foot ulcer	40 (38.4%)
Bedsore	20 (19.2%)
Venous ulcer	16 (15.3%)
Traumatic non-healing chronic ulcer	24 (23.07%)
Others (postoperative wounds, Hansen’s disease, unknown cause)	4(3.8%)
TREATMENT OF DIABETES	
Oral hypoglycemic agents	56 (66.6%)
Insulin	28 (33.3%)

**Figure 2:**
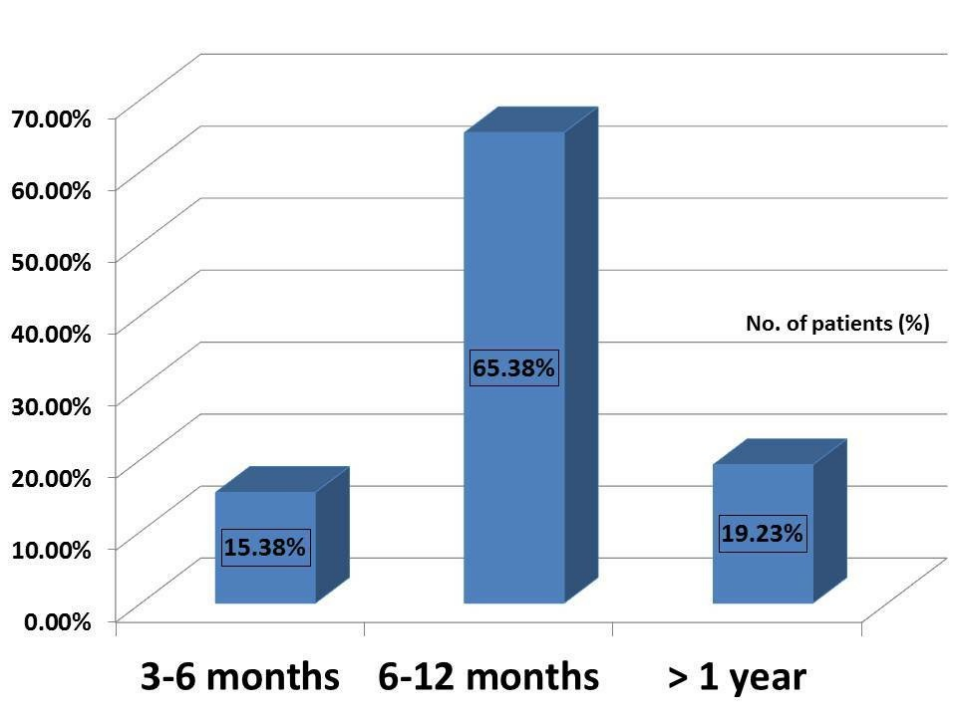
Distribution of chronic non-healing ulcers in the study population

Depending on the duration of the ulcers, they were again classified into the < six months, six to 12 months, and > one-year category, which showed 15.38% (16 patients), 65.38% (68 patients), 19.23% (20 patients), respectively. Since this study was on chronic ulcers, all ulcers of > three-month duration were included in the study (Figure 2).

**Figure 3:**
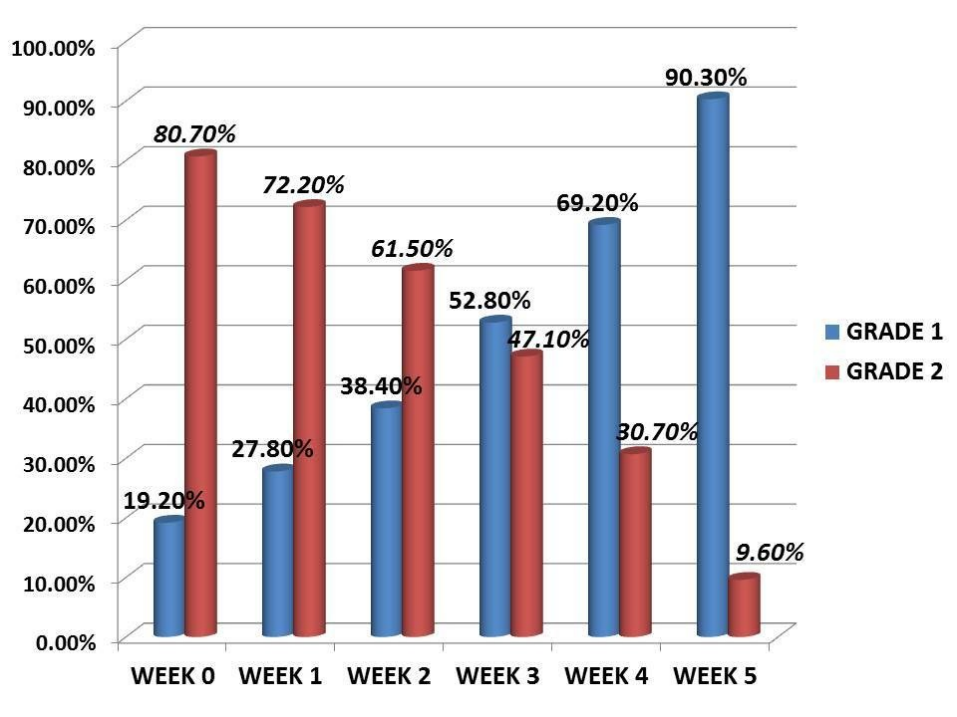
Grade of ulcer - changing pattern in the study period

Based on the grading of the ulcer, they were categorized into Grade 1 and Grade 2 (Figure 3).

**Figure 4:**
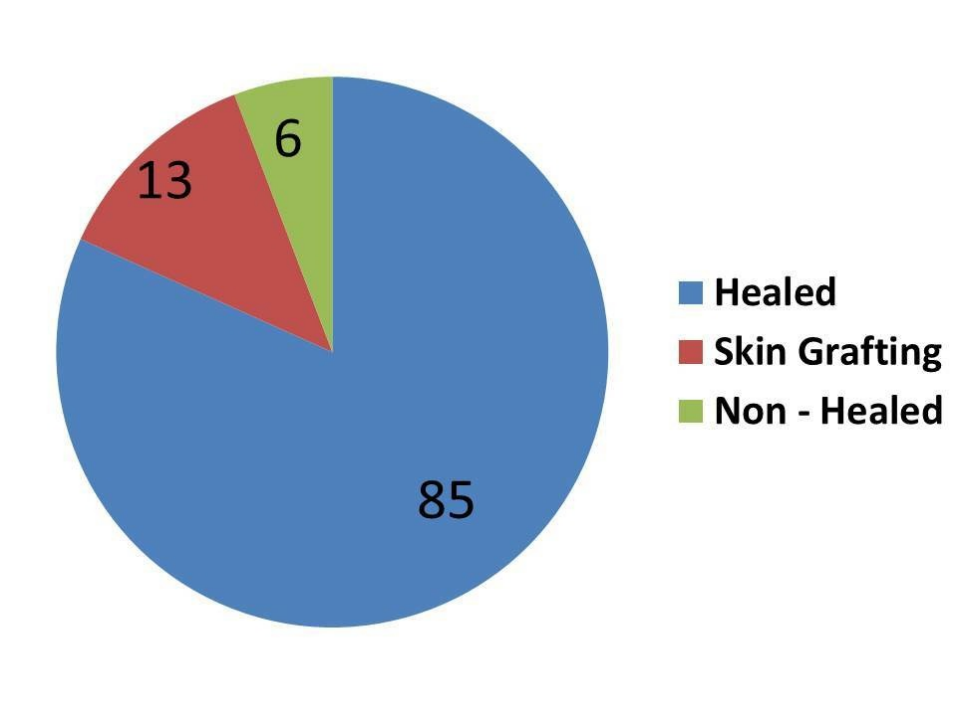
Effect of platelet-rich plasma (PRP) dressing in non-healing ulcers

The healing rates of the ulcers were monitored in weekly intervals till the fifth week. Among 104 patients, 85 patients (81.7%) showed complete healing with a > 75% reduction in wound size and 13 patients (12.5%) showed 50%-75% wound size reduction and went in for wound skin grafting while six patients (5.7%) showed no healing or reduction in wound size (Figure 4).

**Figure 5:**
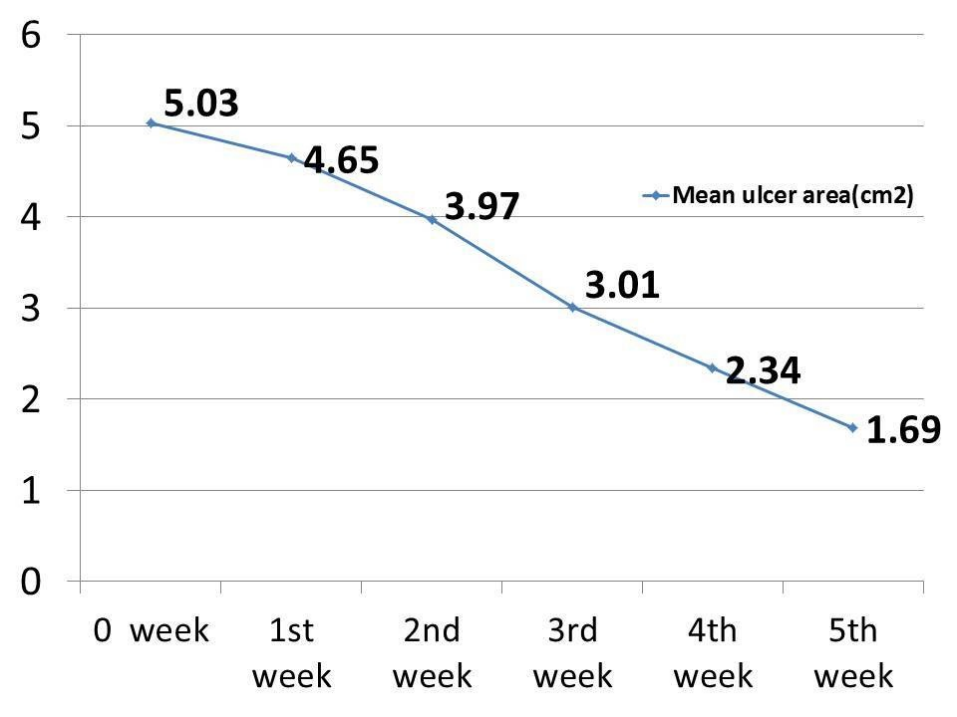
Effect of platelet-rich plasma (PRP) dressing in non-healing ulcers

The healing of the ulcer varied from two months to two years. Among those cases, 81.73% (85 patients) healed ulcers were noted at the end of the last visit. The effectiveness of PRP dressing was evaluated in terms of complete wound healing or > 75% reduction in surface area from the baseline (5.03 cm^2^). For each visit, there was a reduction in the ulcer area. In the last visit, the mean ulcer area became 1.69 cm^2^,^ ^which was significant in this study. The reduction in the mean ulcer area directly corresponded with the number of dressings. There was a significant reduction in the mean ulcer surface area in the fifth week (5.03 vs. 1.69) when compared with the baseline value (Figure 5).

**Figure 6:**
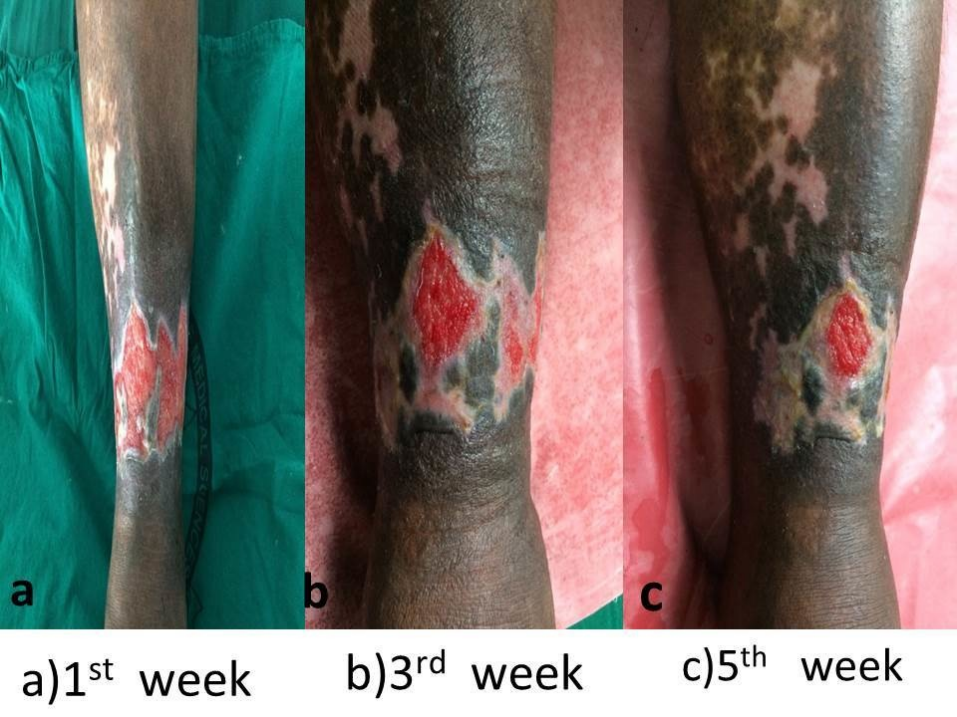
Effect of platelet-rich plasma (PRP) dressing on ulcer surface area (cm²)

The PRP dressings in the third week and fifth weeks were compared with the first week of PRP dressing (Figure 6).

There was a strong reduction in wound discharge and an improvement in granulation tissues within two sittings of dressing due to the antimicrobial properties of PRP. No side effects were noted in the study period.

**Figure 7:**
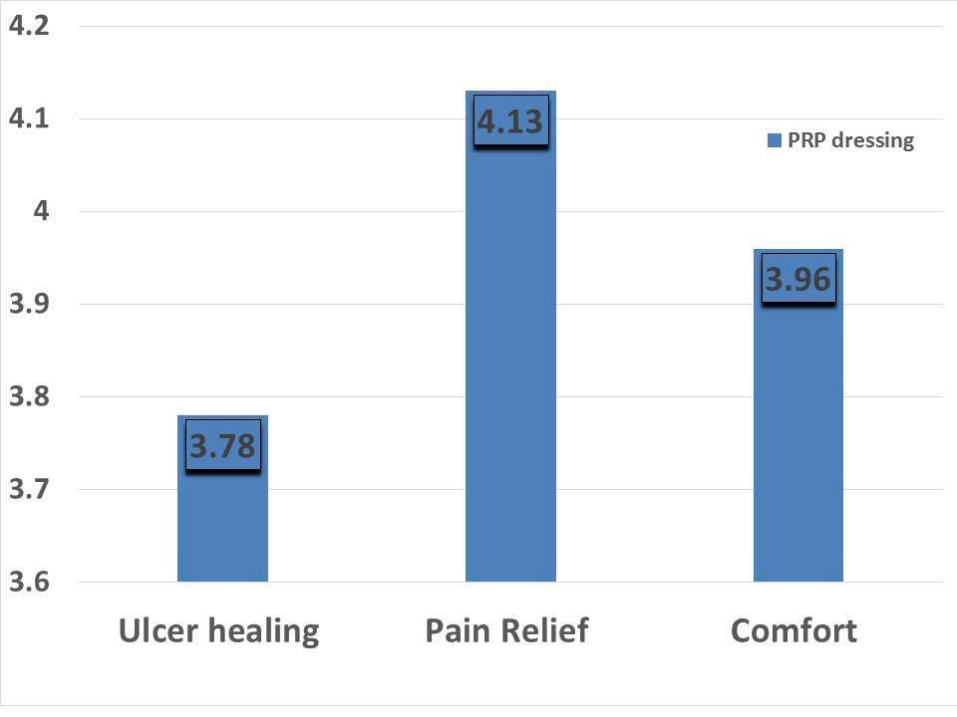
Mean value of patient satisfaction scores for pain relief, healing, and comfort of dressing

The patient satisfaction was measured using the Likert scale for three individual parameters - pain relief, the comfort of the device, and ulcer healing. All three parameters, pain relief (mean score 4.13), comfort of the dressing (mean score 3.96), and ulcer healing (mean score 3.78) were assessed in PRP dressing patients. The overall patient satisfaction was more in the PRP dressing (Figure 7).

## Discussion

The most common causes of non-healing ulcers are GF abnormalities. They are often difficult to treat. Conventional therapies, such as dressings, surgical debridement, and even skin grafting, cannot provide satisfactory healing since these treatments are not able to provide the necessary GFs to modulate the healing process [6]. During the healing process, two sources are vital in wound repair: platelets and wound macrophages. Platelets are considered a rich source of GFs. The anti-inflammatory factors in PRP play a role in wound healing because of the presence of leukocytes, which are at high levels in PRP [6]. Platelets release platelet-derived growth factor (PDGF), platelet-derived angiogenesis factor (PDAF), platelet-derived epidermal growth factor (PDEGF), and platelet factor 4. PDGF is a potent fibroblast mitogen and chemoattractant, which when purified has no endothelial cell chemoattractant activity [7-9]. PDAF causes new capillary formation from the existing microvasculature [10]. In addition to the GFs, platelets release other substances, such as fibronectin, vitronectin, and sphingosine 1-phosphate, which play a vital role in wound healing.

An advantage of PRP over the use of single recombinant human GF is the release of multiple GFs and the differentiation of factors upon platelet activation [11]. It also acts as a barrier to prevent bacterial invasion into the wound and the GF enhances wound healing, but the application of PRP (with good amounts of GFs) gives a better result [12-13]. PRP enriches the wound by multiple growth factors for cell migrations and neo-angiogenesis while PPP contains nutrients for healing [9].

In contrast to this study, many studies highlighted autologous PRP use. Driver et al. carried out the first prospective, randomized, controlled multicenter trial regarding the use of autologous PRP for the treatment of diabetic foot ulcers [11]. While Mehta et al. were successful in healing a chronic lower extremity wound with the use of autologous PRP [14]. Margolis et al. conducted a retrospective cohort study that showed the effectiveness of platelet release (PR) in the treatment of diabetic neuropathic foot ulcers [13]. Crovetti et al. published a prospective study regarding the efficacy of platelet gel (PG) in healing cutaneous chronic wounds [15]. In the present study, by the end of five weeks, 85 patients (81.73 %) showed complete healing while 13 patients (12.5%) needed skin grafting.

In this study, among 104 patients treated with PRP, ulcers in 98 patients (94.23%) showed a good healing rate. The mean surface area of the ulcer was consistently reduced over a period of five weeks. We also noticed a decrease in pain along with healing. This study was only concentrated on homologous PRP dressing, and it was compared with the results of similar studies. This study was mainly done for patients who were not fit for blood donation.

The major limitation of our study was the short follow-up period. A longer follow-up period is required to assess whether the wound healing progresses at a similar rate. Other parameters like the number of debridements required in each week, quality of life, and cost of the total treatment were not studied. The full impact of diabetes on wound healing has not been adequately assessed since this study included non-diabetic cases also. This study did not compare PRP dressing with other dressing material/saline dressing. This study was not attempted in patients with higher grades (Wagner’s grade III, IV and V) of non-healing ulcers.

## Conclusions

Due to the lack of necessary GFs in chronic non-healing ulcers, PRP is a safe and cost-effective method of enhancing healing rates, with no adverse effects. It also reduces the overall hospital stay and morbidity. This study concluded the effectiveness of homologous PRP dressing in the management of chronic non-healing ulcers. According to recent evidence, no study was reported on homologous PRP use. This study is mainly done for patients who are not fit for blood donation.

## References

[1] Pannier F, Rabe E (2013). Differential diagnosis of leg ulcers. Phlebology.

[2] Vivek GK, Rao BHS (2009). Potential for osseous regeneration of platelet rich plasma: a comparative study in mandibular third molar sockets. J Oral Maxillofac Surg.

[3] Parnell LKS (2011). Protein degradation and protection observed in the presence of novel wound dressing components. J Funct Biomater.

[4] Maria-Angeliki G, Alexandros-Efstratios K, Dimitris R, Konstantino K (2015). Platelet-rich plasma as a potential treatment for noncicatricial alopecias. Int J Trichol.

[5] Suryanarayan S, Budamakuntla L, Sha Khadri SI, Sarvajnamurthy S (2014). Efficacy of autologous platelet-rich plasma in the treatment of chronic nonhealing leg ulcers. Plast Aesthet Res.

[6] Ofosu FA (2002). The blood platelet as a model for regulating blood coagulation on cell surfaces and its consequences. Biochemistry (Mosc).

[7] Grotendorst GR, Martin GR, Pencev D, Sodek J, Harvey AK (1985). Stimulation of granulation tissue formation by platelet-derived growth factor in normal and diabetic rats. J Clin Invest.

[8] Knighton DR, Hunt TK, Thakral KK, Goodson WH (1982). Role of platelets and fibrin in the healing sequence: an in vivo study of angiogenesis and collagen synthesis. Ann Surg.

[9] Samani MK, Saberi BV, Tabatabaei SAM, Moghadam MG (2017). The clinical evaluation of platelet-rich plasma on free gingival graft's donor site wound healing. Eur J Dent.

[10] Kim DH, Kim CD, Lee YH, Seo YJ, Lee JH, Lee Y (2011). Can platelet-rich plasma be used for skin healing? Evaluation of effects of platelet-rich plasma on human dermal fibroblast. Ann Dermatol.

[11] Driver VR, Hanft J, Fylling CP, Beriou JM (2006). A prospective, randomized, controlled trial of autologous platelet-rich plasma gel for the treatment of diabetic foot ulcers. Ostomy Wound Manage.

[12] Lacci KM, Dardik A (2010). Platelet-rich plasma: support for its use in wound healing. Yale J Biol Med.

[13] Margolis DJ, Kantor J, Santanna J, Strom BL, Berlin JA (2001). Effectiveness of platelet releasate for the treatment of diabetic neuropathic foot ulcers. Diabetes Care.

[14] Alsousou J, Thompson M, Hulley P, Noble A, Willett K (2009). The biology of platelet-rich plasma and its application in trauma and orthopaedic surgery: a review of the literature. J Bone Joint Surg Br.

[15] Crovetti G, Martinelli G, Issi M (2004). Platelet gel for healing cutaneous chronic wounds. Transfus Apher Sci.

